# Importing Automated Management System to Improve the Process Efficiency of Dental Laboratories †

**DOI:** 10.3390/s20205791

**Published:** 2020-10-13

**Authors:** Cheng-Jung Yang, Ming-Huang Chen, Keng-Pei Lin, Yu-Jie Cheng, Fu-Chi Cheng

**Affiliations:** 1 Program in Interdisciplinary Studies, National Sun Yat-sen University, Kaohsiung 80424, Taiwan; cjyang0521@mail.nsysu.edu.tw; 2 Institute of Manufacturing Information and Systems, National Cheng Kung University, Tainan 70101, Taiwan; p98081027@ncku.edu.tw; 3 Department of Information Management, National Sun Yat-sen University, Kaohsiung 80424, Taiwan; 4 Department of Dental Technology, Shu-Zen Junior College of Medicine and Management, Kaohsiung 82144, Taiwan; chelsea800103@ms.szmc.edu.tw; 5 Fu Chi Dental Laboratory, Kaohsiung 80253, Taiwan; chengfuchi@gmail.com

**Keywords:** prostheses, dental laboratory, productivity 4.0, Internet of things, expert system, RFID

## Abstract

Dental laboratories require manpower resources for manufacturing prostheses and inventory management. In this paper, we developed an automated inventory management system for dental laboratories to improve the production efficiency. A sensing system was developed based on the framework of Internet of things to collect the information of cobalt-chromium disks both in the storage room and manufacturing area, and an expert system was developed to automatically conduct inventory management based on the established rules. The proposed system can reduce the time of recording data and also assist the manager in configuring and managing material orders. The experimental results showed that a large amount of working time is reduced, resulting in the benefits of saving money and improving efficiency in dental manufacturing.

## 1. Introduction

Aging is the main cause of tooth defects. According to a survey by the Department of Health, Taiwan, the five-year accumulative tooth loss rate of the population above 45 and 65 years was 35.9% and 47.2%, respectively. These values indicate that the rate of tooth loss rapidly increases with age, which is the most significant reason for the rapid growth of the dental market [1]. In 2012, the global net worth of the dental device market reached US$17.79 billion, and the mean annual compound growth rate was 6%. Dental devices can be classified into supplies, usual dental equipment, and dental treatment equipment. The global net worth of dental supplies was approximately US$7.94 billion, thus accounting for 47.5% of the overall dental devices. Dental bridges, crowns, artificial dental implants, and dental corrections were the top products used among all dental supplies and accounted for 60% of all dental devices [1]. In 2012, there were more than 140 dental laboratories that manufactured 86.5 million prostheses in Taiwan [2], and the numbers of dental laboratories and prostheses produced have also increased recently, demonstrating the strong potential in the dental device market of Taiwan.

In Taiwan, oral treatment is conducted by certified dentists, and prostheses are manufactured by dental technicians. In every dental laboratory, dentists must cooperate with dental technicians to manufacture prostheses that can perfectly fit patients. To fabricate a perfectly fitted denture, the elements of design, manufacturing process, quality control process, and staff efficiency should be improved. The concept of digital dentistry has been applied to the design of dentures. Digital images can be created using X-ray or three-dimensional (3D) oral scanners and then transformed using dental design software. The denture design and the occlusal simulation can be evaluated, and finally a prosthesis can be fabricated using the tooth carving system [3]. For the other aspects, technologies should be upgraded to attain the goals of processing automation, minimization of the need for manpower, and intelligent design.

Most of the dental laboratories in Taiwan currently are small-scale traditional laboratories in which considerable manpower is required. In the future, the lack of manpower looms as a major problem. Therefore, the technology improvement is necessary. “Industry 4.0” was proposed in Germany and can be a good example of the pursuit of technology improvement [4]. Industry 4.0 is a term that refers to how Germany imported machinery of various original sensors to collect information on factory machinery and then conducted data analysis on the collected information to understand the operations of a plant. When a product is manufactured, the product has different requirements depending on the different characteristics of the product and customers’ demands. However, by analyzing the demand of different products, the concept of “smart manufacturing” can be implemented. The smart manufacturing method can reduce costs and increase the value of goods [5]. Gaub proposed that additive manufacturing, injection molding, and the technologies of Industry 4.0 can be integrated to individualize large-scale products to create single-unit batches in a cost-effective manner [6].

Chen et al. utilized radio frequency identification (RFID) and cloud technology to strengthen the logistics service of a management system. This system can effectively lower costs and enable auxiliary tracking [7]. Lian et al. combined ZigBee and Wi-Fi into one system using Arduino [8]. This system can remotely monitor the changes in temperature and humidity at home without using a computer. Moreover, the system effectively reduced the cost of the monitoring equipment and data transmissions based on the different sensing signals. Zhong et al. used an RFID-enabled real-time manufacturing execution system for mass-customization production shop floor management, including real-time data collection, scheduling, and work-in-progress tracing and tracking [9]. Velandia et al. demonstrated the feasibility of using an RFID system for manufacturing and assembling crank shafts. Data obtained using RFID tags and stored in a server could be integrated with the business to facilitate seamless integration within the factory [10].

Kusiak and Chen reviewed a study and application of expert systems in production planning and scheduling. They identified the relationship between expert systems and operations research approaches and explored the integration of operations research and expert system techniques [11]. Efstathiou et al. used the expert system as a mediator in a computer program to investigate the decision-making complexity of a manufacturing system under different operating characteristics and system layouts [12]. Kłosowski and Gola proposed a model that incorporates fuzzy logic to support managers conducting cost estimations [13]. Bhatt and Buch reviewed studies using rule-based and expert systems in various manufacturing fields. These two methods have considerable potential to enable users to better predict outputs, achieve easy operations, and reduce human error in the manufacturing process [14]. Kumar also identified the trend of using a knowledge-based expert system in manufacturing planning. His method focused on developing a robust system in decision making to handle complex engineering problems [15]. Gramajo and Martinez used the decision support system method to manage network traffic between users and organizations [16]. Decisions and recommendations could be processed by rating a database through a decision tree in models.

Such operations-enhancing activities undertaken by industry are in line with the policies of “Productivity 4.0” that was proposed by the current Taiwan government [17]. The Productivity 4.0 program of Taiwan has promoted three key points. First, technology development has been promoted using Taiwan’s existing technological advantages in electronics, information, and communication industries. The computation and communication networks have been integrated through computation power, and computation and control methods. Functional development in manufacturing, agriculture, and other fields has increased industrial competitiveness. Second, attention has been paid to the structure of small- and medium-sized enterprises in Taiwan to assist them with low resources, and to promote industrial transformation to increase market competitiveness. Finally, human resources have been increased. Although robots are used in Industry 4.0 to reduce the need for human resources, the trend of human–computer interaction will likely continue. It is important to increase control and management capabilities to meet the needs of the future.

Many projects have tried to introduce Productivity 4.0 and focused on discussing the potential benefits and influence of Productivity 4.0 on business management or production lines [18,19,20,21]. Some studies have described the effects after actual implementation. Li developed an automatic grinding wheel dressing and intelligent grinding monitoring system for slanting cylindrical grinders [22]. The processing parameters and results could be saved using MySQL and transmitted to cloud servers using PHP webpages. A corresponding mobile app was also developed. Chen designed a HEXA robot that can be controlled using a man–machine interface [23]. The parameters of this robot can be sent to the Internet by using the network communication module. Wang designed an automated production line by integrating mechanical and electrical equipment with HIWIN industrial robots, programmable logic controllers, Da modules, stepping motor conveyor belts, a mechanism design, and a man–machine interface [24]. Cheng used sensors to develop a framework of Internet of things (IoT) in a storage space to collect data concerning materials and environmental parameters [25]. He then tried to analyze the collected data to explore possible strategies for improving the management efficiency of an inventory.

In this study, an automated management system was developed for use in a dental laboratory. On the basis of an IoT framework, this study established a sensing system to collect information concerning cobalt-chromium (CoCr) disks located in storage and manufacturing areas. Inventory management was automatic by using the rules established by the expert system approach. This technique can reduce the time required by staff to record data, and managers can be reminded to configure and manage material orders through an automated warning system. Thus, the goal of achieving intelligence through automation can be implemented in dental manufacturing. The abbreviations used throughout the paper are summarized in Table 1.

## 2. Materials and Methods

**Figure 1:**
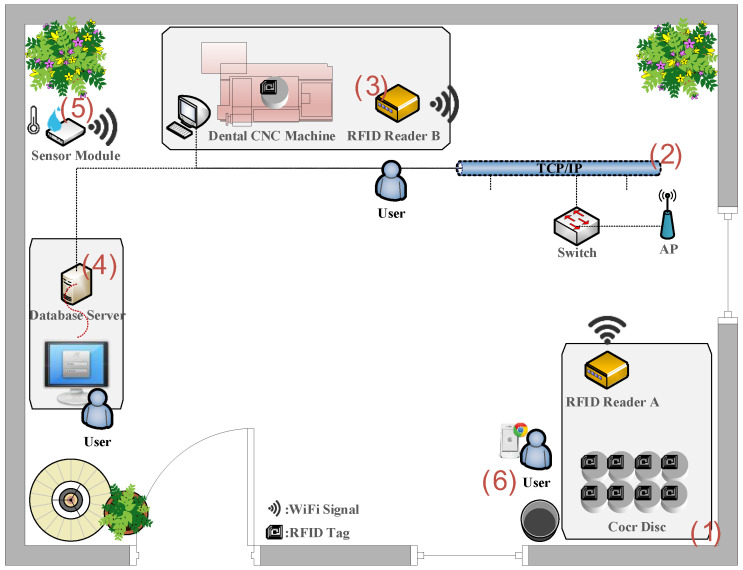
The system architecture of the dental laboratory automated management system.

In this study, RFID and Wi-Fi technologies were used to construct a wireless identification transmission network, and an expert system was used to estimate the inventory of CoCr disks in the storage area. As a result, the automated management system of the dental laboratory was realized. The system architecture consists of six processes, as shown in Figure 1. (1) The user picks up a CoCr disk with an RFID tag in the storage area and then approaches to RFID station A to complete the inbound–outbound inventory operation. (2) RFID station A transmits the inventory data to the access point through Wi-Fi and then transmits the data through the network switch using TCP/IP. The final inventory data are transferred to the database server for storage and update. (3) The user performs a scanning action with RFID to calculate the number of teeth before processing an operation. After collecting the data, RFID station B transmits the data to the database server for storage and upload over Wi-Fi. (4) The database server stores and reads information concerning the inventory of the CoCr disk. Users are instantly informed of the number of discs in inventory, normal inventory, and safety inventory through a human–machine interface. (5) The sensor module collects the temperature and humidity values of the stock area and transmits the data to the ThingSpeak cloud webpage via Wi-Fi. (6) The user observes the current temperature and humidity data and the historical data through a mobile device or a networkable device. A warning is given when the temperature or humidity exceeds the set value.

### 2.1. System Framework

**Figure 2:**
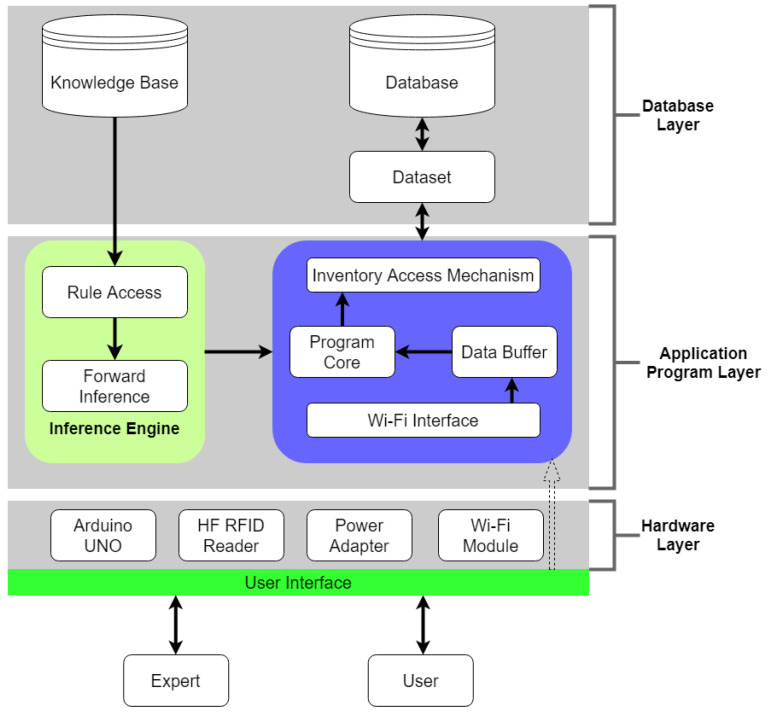
The three-layer framework of the material inventory management system.

The framework of the automated management system can be divided into two sections: the framework of material inventory management and the framework of the sensing node. Both sections use Wi-Fi for the communication network and can be flexibly expanded to extra nodes. As illustrated in Figure 2, there are three layers in the framework of material inventory management—database, application, and hardware. The database layer contains the knowledge base and the database. The rules of material preparation obtained from the expert knowledge system are saved in the knowledge base. The database is a type of relational database. The data include the current inventory, normal inventory, and safety inventory of CoCr disks of different sizes. The well-defined objects and the selected values are all saved in the database. Data storage and modifications are conducted by mapping the dataset category in the database to the memory block. This helps reduce the burden on the system entailing accessing or retrieving data and increases the flexibility of system processing when the database is in the offline state.

The application layer was developed by Microsoft .NET Framework 4.5. Initially, a reasoning engine block was planned for accessing knowledge rules, and then new facts of accessing the inventory were created by matching facts with the use of forward link method. Decisions are made after the new facts fire the rules. The inventory data from the hardware layer are transmitted via the Wi-Fi interface, saved in the data buffer, and then uploaded to the program core every 100ms. The program core then processes both the inventory data from the hardware layer and the results of the forward link calculation. Finally, the inventory access mechanism uses the methods and properties provided by the dataset category, which are in line with the properties of flexible expansion and program code reuse. In the hardware layer, by using a 12V DC transformer, automated material management is conducted by combining an Arduino UNO control board and a high-frequency (HF) RFID. The data are transmitted using the Wi-Fi module. In the framework of the sensing node, the Arduino UNO control panel is the core that connects the modules of temperature and humidity. After controller processing, the retrieved data are packed and then transmitted via Wi-Fi to the ThingSpeak website for storage and presentation of inventory environmental information.

### 2.2. Process of the Expert System

Newell and Simon proposed a production system model that became the foundation of rule-based expert systems [26]. Experts are those who have knowledge and experience in a specific area. When setting up a knowledge system, it is crucial to know how to solve a problem before making a machine to solve this problem. Therefore, it is critical to establish a framework by applying the thinking process of experts to the computer program. Then, a knowledge base with the if–then structure should be defined by transforming expert knowledge into facts and rules. Finally, a system that can solve problems in a specific area can be made intelligent. 

The basic structure of this system, as shown in Figure 2, includes a knowledge base, database, reasoning engine, interpretation tool, and user interface. The knowledge base contains knowledge that can be used to solve problems in specific areas and is composed of rules that combine objects and values using the if–then structure. The database stores a set of facts and compares these facts with the “if” part of rules in the knowledge base. The reasoning engine loads rules in the knowledge base and facts in the database to reason and obtain solutions. The interpretation tool provides users with concrete conclusions and reasoning evidence from the system. The user interface is the bridge between users and the expert system. 

**Figure 3:**
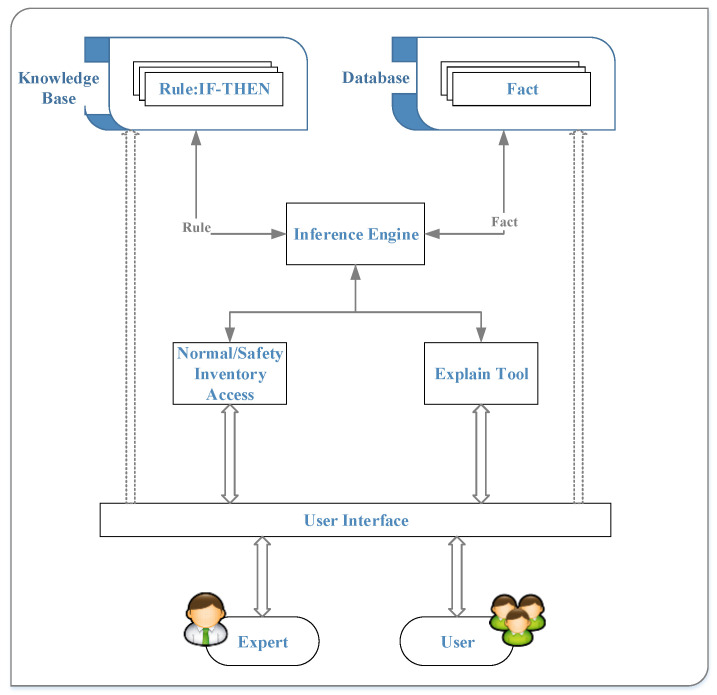
The components and operation processes of the expert system.

This system uses the CLIPS expert system software [27] to verify all processes of rule firing and face matching and to ensure that the expert system can be integrated into the material inventory management system. The process of the expert system in this study is illustrated in Figure 3. The inference engine is the main unit of the system and serves as the brain of a virtual agent. After retrieving the material preparation rules from the knowledge base and the facts in the database, the normal and safety inventory numbers are obtained by conducting sequential fact matching and rule firing through the forward reasoning method. Consequently, every cycle in the inference process and the new facts are the interpretation tools for verifying the inference results. Then, the reasoning and the decision-making process of the virtual agent is completed, and finally, the users or experts can access the material preparation information that assists decision making via the user interface. Finally, the goal of decision support can be achieved.

**Figure 4:**
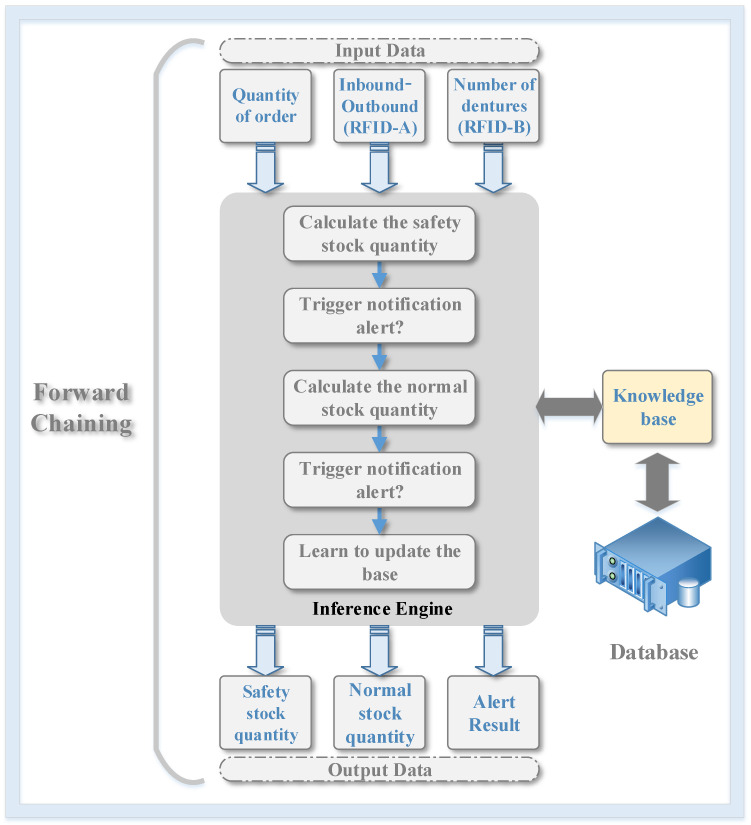
The architecture of the expert system.

The architecture of the expert system is shown in Figure 4. The inference engine is forward chaining. When the order is received, or there are inbound/outbound works in the storage room sensing by the RFID reader A, or there are changes in the number of dentures in the manufacturing area sensing by the RFID reader B, the inference engine will be activated to fire the corresponding rule in the knowledge base. There are five steps in the inference engine. The first step calculates the safety stock quantities of CoCr disks in different sizes, and the second step determines whether to trigger the alert of required safety inventory based on the result of the first step. The third step calculates the normal stock quantities of CoCr disks in different sizes, and the fourth step determines whether to alert of normal inventory or not. Finally, the fifth step updates the data and rules to the database and knowledge base and displays the alert result and safety and normal stock quantities of CoCr disks in different sizes to the human–machine interface.

### 2.3. Hardware Development

#### 2.3.1. Hardware Framework of Material Inventory Management.

**Figure 5:**
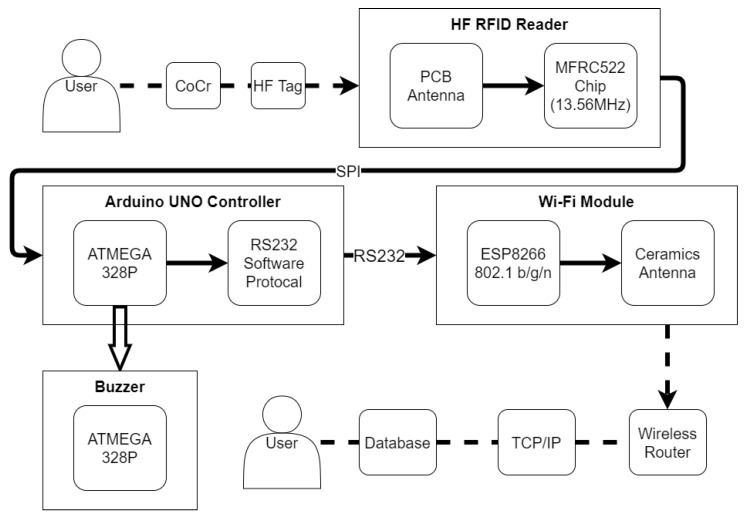
The hardware framework of material inventory management.

The hardware framework of material inventory management is illustrated in Figure 5. The Arduino UNO controller panel, which is the core of the system, communicates with the 13.56 MHz HF RFID reader using the SPI interface. When the CoCr disks are transported in or out of the storage room for CNC (computer numerical control) manufacturing, the HF RFID tag on each CoCr disk is scanned using the HF RFID reader. The HF RFID tag is a passive tag. When the tag receives the signals from the HF RFID reader, the ID of the tag is read by the HF RFID reader and then transmitted to the controller panel to be saved. The controller then activates the buzzer by transmitting an electrical signal.

The controller transmits the scanned ID of the tag to the Wi-Fi module through RS232 communication agreement every 500 ms. The Wi-Fi module can work under the access point mode or station mode. When the Wi-Fi module receives the data, they are packed and transmitted further to the AP mode wireless router through the ceramic antenna and then saved to the computer database via the TCP/IP network. The app of the man–machine interface in the computer is developed using Visual C++. The database is deployed with the SQL Server 2014 Express.

#### 2.3.2. Hardware Framework of Sensing Nodes

**Figure 6:**
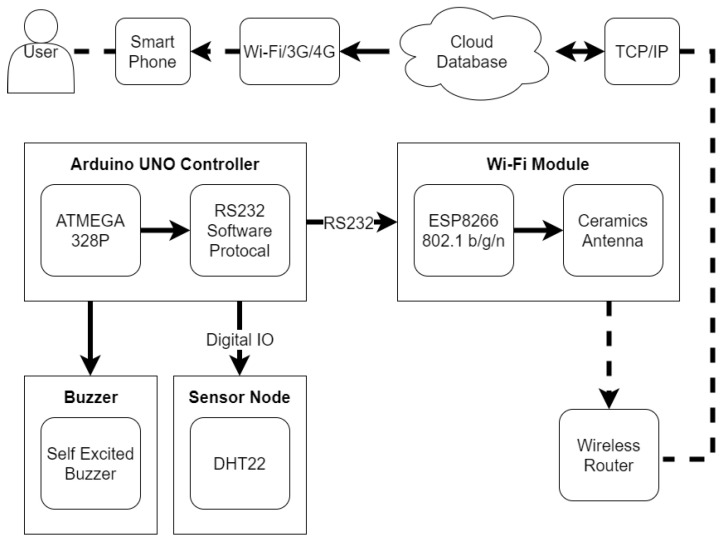
The hardware framework of the sensing node.

The hardware framework of the sensing node is displayed in Figure 6 and is modified from the hardware framework of material inventory management by adjusting the input and output unit. The input unit contains a DHT22 temperature and humidity sensor that detects the environmental parameters of the storage room [28]. These parameters are processed into valuable information by the Arduino microcontroller and then transmitted via Wi-Fi to the output unit. The ThingSpeak cloud database is the core in the output unit. Users can obtain the curves and the statuses of the temperature and humidity in the storage room using mobile devices. The system sounds an alarm if the environmental parameters exceed the safety threshold.

## 3. Results

### 3.1. System Practice and Verification

**Figure 7:**
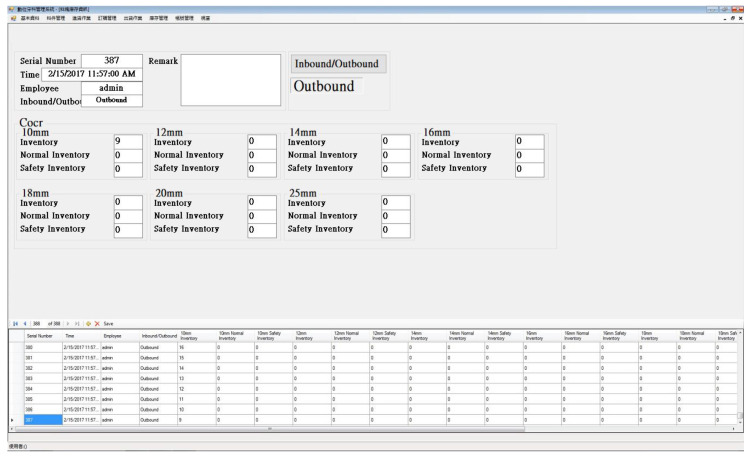
The human–machine interface of the material inventory system.

The information about the inbound and outbound operations and the entire CoCr disk inventory, including disk size, current inventory, normal inventory, and safety inventory, is displayed in the human–machine interface of the material inventory system. The human–machine interface of the material inventory system is illustrated in Figure 7. There are seven categories of disk sizes: 10, 12, 14, 16, 18, 20, and 25 mm disks. The experimental group used the newly developed system for recording the CoCr disk inbound and outbound operations of the stock area, and the control group used currently existing manpower to record the CoCr disk inbound and outbound operations of the stock area. The timing of the inbound and outbound operations that used the CoCr disks of the control and experimental groups was compared to verify the effectiveness of the new system.

**Figure 8:**
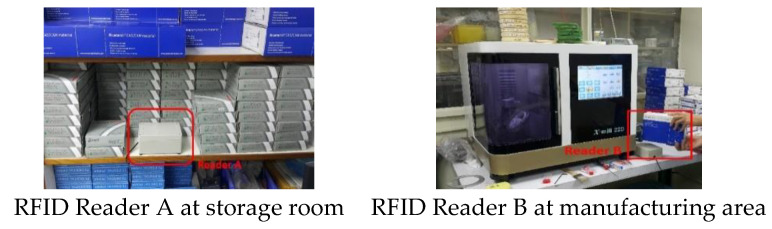
The RFID readers at storage room and manufacturing area.

The testing was conducted in a dental laboratory. After testing, the operational sensing distance of the RFID reader is approximately 2 to 3 cm at all sites, including the storage room (the Reader A shown in Figure 8) and the manufacturing area (the Reader B shown in Figure 8). The scanning and recording of the inbound and outbound work could be completed within 2 s. The comparison between the inbound–outbound storage time for a single disk of the control and experimental groups is presented in Table 2. The average time of the control group was 48 s, which was considerably longer than that of the experimental group, 6.9 s. Therefore, inventory counting can be performed faster using the newly developed system. The efficiency was improved approximately 7 times, and the recording errors caused by the staff could be avoided.

**Figure 9:**
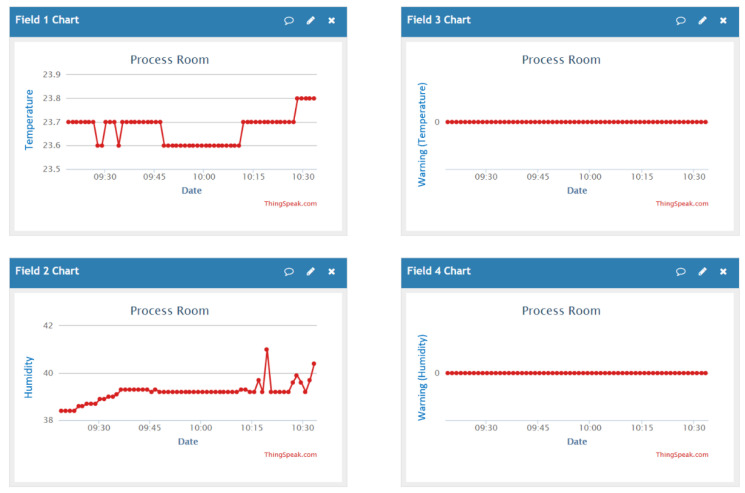
Temperature and humidity curves and alarm curves recorded on the ThingSpeak website.

The testing site for temperature and humidity was located next to the CoCr disk storage area. The upper limit of temperature was set at 26 °C and that of humidity was set at 45% relative humidity. The system sounds an alarm if the detected temperature or humidity exceeds the upper limit. The values of temperature and humidity displayed on the ThingSpeak website are shown in Figure 9. In both temperature and humidity curves, 200 sets of values are displayed, which were recorded every 20 s. The temperature varied slightly because of the activities of humans inside the room but was nevertheless maintained at approximately 24 °C. Humidity was maintained at approximately 40% relative humidity. Both values were lower than the upper limit and the value was always zero (normal status) and never one (abnormal status) in both temperature and humidity alarm curves on the right side.

### 3.2. Experimental Results of the Expert System

After discussing with the dental technicians and the technical director of the dental laboratory, 26 rules were created in the expert system, as shown in Table 3. The system then updated the normal inventory and underwent warning processes to determine the safety inventory by using these rules. In rules 1–6, the safety inventory was set based on whether orders are present or not. In rules 7–12, a warning sign was sent to users for further material preparation if the actual inventory was smaller than the specified safety inventory. In rules 13–18, a new normal inventory was calculated by dividing the number of ordered prostheses by the base number, rounding the result to the nearest whole number, and finally adding the normal inventory amount to the result. In rules 19–24, a comparison was conducted between the actual inventory and the normal inventory. If the actual inventory was smaller, then warning signals were sent to users for further material preparation. In rule 25, the amount of processed prostheses scanned at site B was added to the historical data in the database. Then, a new maximum number of prostheses that can be processed in the new single disk was calculated. In rule 26, the new maximum number to be processed was compared with the old one. If these two numbers are not equal, the new number will replace the old one and the corresponding base number of disks of different sizes will be updated.

The experimental results are presented in Table 4, which is the required safety inventory. The normal inventory was calculated by dividing the number of orders by the base number, rounding the result to the nearest whole number, and finally adding the result to the safety inventory. The current inventory presented in Table 4 is sufficient for all denture-processing orders. As long as the normal inventory is maintained, materials are prepared in time, and the loss due to human involvement is reduced in the dental laboratory.

## 4. Discussion

The inventory management for protheses manufacturing of small- and medium-sized dental laboratories heavily relies on manual recordings and the manager’s personal experiences. Manual recordings are prone to errors, and the manager’s personal experiences may not be able to be inherited by new managers. Therefore, we introduced the information system based on IoT and RFID and built an expert system based on the manager’s experiences. The ease of deployment and stability of the expert system fit well to the intelligent manufacturing of small- and medium-sized dental laboratories. In the knowledge base of the expert system, there are 26 rules derived based on the interview with a domain expert, which automates the warnings of required safety inventory and the updates of the normal inventory.

In this study, experiments were conducted on one type of material, CoCr disks, with different sizes. According to the data of the experimental and control groups, during a one-month period where there are around 350 cases, around 14,385 s are conserved. Over the course of an entire year, about 48 h are conserved. Assuming human efforts were compensated at the minimum wage in Taiwan in 2020 which is 158 Taiwan dollars [29], this time savings could save around 260 U.S. dollars. This estimate is only for completing a prosthesis fitting using one of the manufacturing processes. According to the administrator of the dental laboratory, a prosthesis fitting usually includes 12 procedures, five for which implementation of the automated management system would be appropriate. Moreover, five types of material are commonly used. If the system estimates a five-year lifespan, approximately 32,500 USD can be saved. It is noted that this does not include other losses caused by manual transcription errors or the inefficiency of follow-up work.

With respect to the storage environment in which materials are stored, some materials are sensitive to changes in temperature and humidity. Once such materials are exposed to an environment unsuitable for their preservation, their material properties may change slightly. This may reduce the life of the prosthesis. Therefore, an environmental control immediately determines changes in a storage space and deals with the situation beyond the storage standard.

Compared to relevant research works, like the work of Chen et al. [8] which also utilized RFID technology to reduce the cost of manpower, we further capitalized on an expert system to learn the inventory management rules to improve the processes of automated management. Another RFID-based manufacturing system proposed by Velandia et al. [11] is for large-scale massive production. The building cost is too high for the manufacturing of prostheses in dental laboratories. Our proposed system possesses the benefits of the ease of deployment as well as low costs for small- and medium-sized dental laboratories.

The material automated management system developed in this study has the advantages of low cost and expansion flexibility. The results revealed that this system effectively reduces the processing time in the dental laboratory. The normal and safety inventories can be calculated in real time by the expert system, which combines the number of operations in the inbound, outbound, and CNC manufacturing processes and the rules based on expert knowledge. Therefore, the manual work loss due to an insufficient quantity of materials or forgetfulness of material preparation can be effectively reduced, which achieves the goal of assisting in support and decision making. Moreover, the changes in the environmental parameters of the storage room are monitored by the cloud platform to increase the stability of inventoried materials. A comparison of with and without the system implementation is shown in Table 5. The test results revealed that this system meets the goals of achieving an industrial upgrade from the traditional dental laboratory and satisfying the framework of Productivity 4.0.

## 5. Conclusions

In this paper, we proposed an automated management system for the inventory management in manufacturing protheses in dental laboratories. The system also features environment monitoring on the material storage, and an expert system was introduced to assist on planning the material orders. The experimental results showed that the working time in the dental laboratories can be significantly reduced. In the future, we plan to improve the system availability on different materials in addition to the CoCr disks, and also the system efficiency. Although preliminary results of predicting the inventory amount using the expert system were attained, the loading time from the rule base increases significantly when the material inventory increases. This can be improved by using the backward link method. Another possible improvement is employee education and training. Currently, all employees have become accustomed to the method of manually recording data. A hasty change in their work habits might lead to resistance. Therefore, implementing a new system requires powerful motivation. The time and cost management benefits can be impressed upon them. Great benefits can be obtained by gaining the trust of the employees and then implementing the proposed automated management system.

## Figures and Tables

**Table 1:** Summary of abbreviations.

Abbreviation	Description
CoCr	Cobalt-chromium
IoT	Internet of things
RFID	Radio-frequency identification
HR	High frequency

**Table 2:** The average inbound/outbound time of a single disc in experimental and control groups.

Average Time(Seconds)	Experimental Group	Control Group
Inbound	3.3	24.2
Outbound	3.6	23.8
Total time	6.9	48

**Table 3:** The rules of the expert system.

Rule Number	IF	THEN
1	No order for single posterior teeth	The minimal (safety) inventory amount of 10 mm discs is set as 3
2	No order for single front teeth	The minimal (safety) inventory amount of 12 mm discs is set as 2
3	No order for three-unit dental bridge	The minimal (safety) inventory amount of 14 mm discs is set as 2
4	No order for multi-unit dental bridge	The minimal (safety) inventory amount of 16/18 mm discs is set as 1
5	No order for dental implant	The minimal (safety) inventory amount of 20 mm discs is set as 1
6	No order for full mouth reconstruction	The minimal (safety) inventory amount of 25 mm discs is set as 1
7	Inventory amount of 10 mm discs < minimal (safety) inventory amount of 10 mm discs	Alarm for further preparation of the discs
8	Inventory amount of 12 mm discs < minimal (safety) inventory amount of 12 mm discs	Alarm for further preparation of the discs
9	Inventory amount of 14 mm discs < minimal (safety) inventory amount of 14 mm discs	Alarm for further preparation of the discs
10	Inventory amount of 16/18 mm discs < minimal (safety) inventory amount of 16/18 mm discs	Alarm for further preparation of the discs
11	inventory amount of 20 mm discs < minimal (safety) inventory amount of 20 mm discs	Alarm for further preparation of the discs
12	inventory amount of 25 mm discs < minimal (safety) inventory amount of 25 mm discs	Alarm for further preparation of the discs
13	Having orders for single posterior teeth	Calculate the normal inventory amount of 10 mm discs: (Order number/base number 1) + minimal (safety) inventory amount
14	Having orders for in single front teeth	Calculate the normal inventory amount of 12 mm discs: (Order number/base number 2) + minimal (safety) inventory amount
15	Having orders for in three-unit dental bridge	Calculate the normal inventory amount of 14 mm discs: (Order number/base number 3) + minimal (safety) inventory amount
16	Having orders for in multi-unit dental bridge	Calculate the normal inventory amount of 16/18 mm discs: (Order number/base number 4) + minimal (safety) inventory amount
17	Having orders for in dental implant	Calculate the normal inventory amount of 20 mm discs: (Order number/base number 5) + minimal (safety) inventory amount
18	Having orders for in full mouth reconstruction	Calculate the normal inventory amount of 25 mm discs: (Order number/base number 6) + minimal (safety) inventory amount
19	Inventory amount of 10 mm discs < normal inventory amount of 10 mm discs	Alarm for further preparation of the discs
20	Inventory amount of 12 mm discs < normal inventory amount of 12 mm discs	Alarm for further preparation of the discs
21	Inventory amount of 14 mm discs < normal inventory amount of 14 mm discs	Alarm for further preparation of the discs
22	Inventory amount of 16/18 mm discs < normal inventory amount of 16/18 mm discs	Alarm for further preparation of the discs
23	Inventory amount of 20 mm discs < normal inventory amount of 20 mm discs	Alarm for further preparation of the discs
24	Inventory amount of 25 mm discs < normal inventory amount of 25 mm discs	Alarm for further preparation of the discs
25	The number of processed prostheses scanned at station B (discs in 10/12/14/16/18/20/25 mm)	Calculate the average amount of previously processed dentures at station B (10/12/14/16/18/20/25 mm disc)
26	The number of previously processed prostheses is not equal to the base number 1–6	Update the base number of 1–6

**Table 4:** Experimental results from the expert system.

Disc Size	Ordered Denture Number	Base Number	Actual Inventory Amount	Normal Inventory Amount	Minimal (Safety) Inventory Amount
10 mm	30	14	6	5	3
12 mm	26	15	10	4	2
14 mm	18	14	5	3	2
16 mm	20	17	3	2	1
18 mm	18	17	3	2	1
20 mm	2	16	2	1	1
25 mm	0	18	2	1	1

**Table 5:** Comparison of with and without system implementation.

	Without the System	With the System
Operation mode	Manual record	Scan sensing by RFID technology
System framework	No	Integration of the computer and the Cloud
Expansion flexibility	No	High
Need for manpower	High	Low
Environmental parameters	Unable to be monitored	Effectively monitored
Operation time	Longer	Shorter
Intelligent level	No	Expert system
Material preparation	Predicted by human	Assisted by the intelligent decision support system
